# Laboratory Toxicity and Field Efficacy of Four Microbial-Derived Pesticides Combined with Two Adjuvants Against *Lygus pratensis* (Hemiptera: Miridae)

**DOI:** 10.3390/insects17070751

**Published:** 2026-07-22

**Authors:** Wei Lu, Ruihao Li, Xiang Yan, Hailong Gao, Yanru Wang, Yubo Jiao, Zongfang Fan, Yujiao Wang

**Affiliations:** 1 Key Laboratory of the Pest Monitoring and Safety Control of Crops and Forests of the Universities of the Xinjiang Uygur Autonomous Region, College of Agronomy, Xinjiang Agricultural University, Urumqi 830052, China; teerakon@sina.com (W.L.); 15556345679@163.com (R.L.); yxxxx0000@163.com (X.Y.); g2389057673@163.com (H.G.); wyr12326@outlook.com (Y.W.); 13963031415@163.com (Y.J.); zfan@xjau.edu.cn (Z.F.); 2 Engineering Research Centre of Cotton, Ministry of Education, Urumqi 830052, China

**Keywords:** *Lygus pratensis*, microbial pesticides, adjuvants, laboratory toxicity, field efficacy

## Abstract

Systematic field data on adjuvant-amended microbial-derived pesticides against *L. pratensis* in arid, high-ultraviolet cotton regions remain scarce. This study systematically evaluated the laboratory toxicity and field efficacy of four kinds of microbial-derived pesticides (abamectin, emamectin benzoate, *B. bassiana*, and *M. anisopliae*) and their combinations with adjuvants against *L. pratensis* in Xinjiang cotton fields. As a comprehensive regional study, this work elucidates the differential enhancement patterns of d-limonene and mineral oil on antibiotic insecticides and entomopathogenic fungi, providing targeted field data for pesticide reduction strategies. The laboratory toxicity of seven microbial-derived pesticides was determined using the leaf-tube residual film method. Four effective agents were selected and combined with d-limonene or mineral oil for field efficacy trials. Abamectin and emamectin benzoate exhibited rapid and high insecticidal activity, with 48 h LC_50_ values of 1.198 mg/L and 3.424 mg/L, respectively. The two entomopathogenic fungi exhibited slower insecticidal activity than the chemical insecticides but maintained relatively stable control effects throughout the observation period. In field trials, when abamectin or emamectin benzoate was applied at a 30% reduced rate in combination with mineral oil or d-limonene, the control efficacy was equivalent to or higher than that of the full-rate application of the pesticide alone. The treatment of abamectin (4.20 g a.i./hm^2^) plus mineral oil achieved the highest control efficacy (89.24%) at 3 days post-treatment. For *B. bassiana* and *M. anisopliae*, reduced-rate adjuvant-amended treatments showed numerically higher initial and residual efficacy than the full-rate single-agent fungal treatments. The treatment of *M. anisopliae* (7.35 × 10^12^ spores/hm^2^) plus d-limonene reached 70.50% efficacy at 7 days post-treatment, which was significantly higher than that of the full-rate fungal treatment alone. No phytotoxicity symptoms were observed on cotton plants. Under the tested conditions, the rational combination of microbial-derived pesticides with appropriate adjuvants demonstrates the potential for a 30% reduction in pesticide dosage while maintaining or improving field efficacy, providing region-specific reference for the sustainable management of *L. pratensis* in Xinjiang cotton fields.

## Simple Summary

In the cotton-growing regions of Xinjiang, China, extensive reliance on conventional insecticides has promoted the development of resistance in *Lygus pratensis*, making its management increasingly challenging. Moreover, excessive pesticide applications have resulted in environmental contamination, pesticide residues, and increased ecological risks. In this study, we evaluated four kinds of microbial-derived pesticides (abamectin, emamectin benzoate, *Beauveria bassiana*, and *Metarhizium anisopliae*) mixed with two adjuvants—d-limonene and mineral oil—for the control of *L. pratensis*. The results showed that a 30% reduction in the application rate of these pesticides, when combined with appropriate adjuvants, achieved control efficacy comparable to or numerically higher than the corresponding full-rate pesticide treatments, with no significant efficacy loss observed. Notably, reduced-rate treatments combined with adjuvants showed numerically earlier efficacy onset for the two fungal biocontrol agents, which normally act slowly. This study provides a feasible and environmentally friendly strategy to reduce pesticide inputs and achieve sustainable management of *L. pratensis* in cotton fields.

## 1. Introduction

Xinjiang is one of the leading regions for high-quality cotton production in China. In recent years, changes in cropping systems and shifts in pest communities have resulted in *L. pratensis* becoming one of the most important piercing-sucking pests during the cotton squaring and boll-setting stages. Adults and nymphs feed on the sap of young cotton tissues, causing bud and boll abscission, leaf and shoot damage, and resulting in substantial reductions in cotton yield and fiber quality [1]. Chemical insecticides have long been the primary method for controlling *L. pratensis*. However, over-reliance on chemical pesticides has led to increased pest resistance, destruction of natural enemies, and environmental pollution [2]. Therefore, the development of effective, low-toxicity, and environmentally sustainable pest management strategies, especially the application of microbial-derived pesticides for alternative or adjuvant-amended control, has become a research priority.

Microbial-derived pesticides play an increasingly important role in integrated pest management (IPM) due to their unique modes of action, safety to non-target organisms, and low risk of resistance. Abamectin and emamectin benzoate are two commonly used microbial-derived antibiotic insecticides with contact and stomach toxicity [3]. Entomopathogenic fungi, including *M. anisopliae* and *B. bassiana*, kill pests via cuticle infection and show high ecological safety [4]. Nevertheless, some microbial-derived pesticides have limitations, such as relatively slow speed of action and high susceptibility to environmental conditions. Studies have shown that the addition of suitable adjuvants such as plant essential oils and mineral oils can significantly improve the bioactivity of pesticides by enhancing wetting, spreading, and penetration of active ingredients, thereby enabling reduced pesticide inputs while maintaining or improving control efficacy [5]. However, the specific integration of these adjuvants must be tailored to the distinct physiological and environmental limitations of different microbial-derived pesticides. For instance, antibiotic insecticides such as abamectin and emamectin benzoate, despite their rapid and high initial toxicity against piercing-sucking pests, are highly susceptible to rapid photodegradation under the intense solar ultraviolet radiation characteristic of the Xinjiang cotton-growing region, which severely shortens their residual control period [6,7]. On the other hand, entomopathogenic fungi like *B. bassiana* and *M. anisopliae* offer persistent and eco-friendly suppression, but their field efficacy is frequently constrained by a delayed knockdown effect and high vulnerability to the arid and low-humidity microclimates typical of Northwest China, which hinders spore germination and cuticle infection [8,9]. However, most existing studies on pesticide adjuvants focus on humid and semi-humid agricultural regions. Research on the adjuvant effects of d-limonene and mineral oil on microbial-derived pesticides under the unique arid, high-ultraviolet conditions of Northwest China is still limited, especially for the mirid pest *L. pratensis*, which lacks systematic field evaluation data. Furthermore, few studies have directly compared the differential adjuvant effects of the same additives on both antibiotic insecticides and entomopathogenic fungi under identical field conditions. Consequently, employing plant essential oils such as d-limonene or mineral oils presents a strategic approach to overcome these respective bottlenecks by shielding UV-sensitive active ingredients and enhancing cuticular penetration for the antibiotics, as well as by improving the moisture retention and adhesion of fungal spores.

This study systematically evaluated the laboratory toxicity and field efficacy of microbial-derived pesticides against *L. pratensis* and further investigated their performance when combined with d-limonene or mineral oil. In this study, we proposed the following working hypothesis: the addition of d-limonene or mineral oil can compensate for the efficacy loss caused by 30% pesticide dosage reduction, and the two adjuvants will show different enhancement patterns for antibiotic insecticides and entomopathogenic fungi due to their distinct action mechanisms. The objective was to screen efficient and safe reduced-application schemes and provide theoretical and practical support for the green and sustainable control of *L. pratensis* in Xinjiang cotton fields.

## 2. Materials and Methods

### 2.1. Test Insects

*L. pratensis* adults were collected from cotton fields in Caoyuanhe New Village, Ayibage Town, Awati County, Aksu Prefecture, Xinjiang, China (40°38′28″ N, 83°39′45″ E) from June to August 2024. The cotton cultivar grown at the collection site was Xinluzhong 82. The collected adults were maintained in a climate-controlled chamber at 25 ± 1 °C, 50 ± 5% relative humidity, and a 16:8 h (L:D) photoperiod.

### 2.2. Test Pesticides

Technical-grade abamectin (99.6%), emamectin benzoate (98.4%), rotenone (65%), matrine (71%), and azadirachtin technical powder (37%), all purchased from Shanghai Aladdin Biochemical Technology Co., Ltd. (Shanghai, China). The entomopathogenic fungi *M. anisopliae* strain CQMa421 (8 × 10^9^ spores/mL) and *B. bassiana* strain ZJU435 (1 × 10^10^ spores/mL) were provided by Chongqing Julixin Bioengineering Co., Ltd. (Chongqing, China). Before the experiment, the conidial germination rate of the two entomopathogenic fungi was determined. Briefly, 100 μL of spore suspension was spread on Sabouraud dextrose agar (SDA) plates (Qingdao Hopebiol Biotechnology Co., Ltd., Qingdao, China) and incubated at 25 °C for 24 h. A spore was considered germinated when the germ tube length exceeded half of the spore diameter. The germination rates of *M. anisopliae* CQMa421 and *B. bassiana* ZJU435 were 92.3% and 90.7%, respectively, which met the experimental requirements. Commercial formulations included 10% abamectin suspension concentrate (SC) and 1.14% emamectin benzoate microemulsion (ME) (Dow AgroSciences LLC, Indianapolis, IN, USA), 5% d-limonene soluble concentrate (SL) (Oro Agri International Inc., San Antonio, TX, USA), and 99% mineral oil emulsifiable concentrate (EC) (SK Enmove Co., Ltd., Seoul, Republic of Korea). Acetone (analytical grade) and Tween-80 (Shanghai Aladdin Biochemical Technology Co., Ltd., Shanghai, China) were used as solvents and emulsifiers during pesticide preparation. For the preparation of test solutions, the required volume of pesticide stock solution and adjuvant was accurately measured according to the target concentration, then added to distilled water and fixed to the final volume. The solution was stirred with a magnetic stirrer for 5 min to ensure uniform mixing before use.

### 2.3. Instruments

The instruments used in this study included RXM-258A climate-controlled chamber (Ningbo Jiangnan Instrument Factory, Ningbo, China), LE204E/02 electronic balance (Mettler-Toledo Instrument Shanghai Co., Ltd., Shanghai, China), Finnpipette^®^ pipettes (Thermo Fisher Scientific Inc., Waltham, MA, USA), microsyringe (Shanghai Anting Micro-injector Factory, Shanghai, China), and LQQ-25L backpack electric sprayer (Taizhou Lvqingting Sprayer Co., Ltd., Taizhou, China).

### 2.4. Laboratory Bioactivity Assay

The leaf-tube residual film method was used to evaluate the toxicity of seven pesticides and their mixtures with two adjuvants against *L. pratensis*. For single-agent treatments, each pesticide was diluted with water to five serial concentrations, with sterile water used as the control. For reduced-rate (30% reduction) plus adjuvant treatments, each pesticide was diluted with water containing 0.50 mL/L adjuvant to five serial concentrations, and water containing 0.50 mL/L adjuvant was used as the control. The concentration ranges were determined based on preliminary bioassays and previous related studies. Five serial concentrations with a 2-fold dilution interval were set for each pesticide, with specific values as follows: Abamectin: (0.625, 1.25, 2.5, 5, 10 mg/L), Emamectin benzoate: (1.25, 2.5, 5, 10, 20 mg/L), Rotenone: (6.25, 12.5, 25, 50, 100 mg/L), Matrine: (12.5, 25, 50, 100, 200 mg/L), azadirachtin: (12.5, 25, 50, 100, 200 mg/L), *B. bassiana*: (3.125 × 10^7^, 6.25 × 10^7^, 1.25 × 10^8^, 2.5 × 10^8^, 5 × 10^8^ spores/L), *M. anisopliae*: (3.125 × 10^7^, 6.25 × 10^7^, 1.25 × 10^8^, 2.5 × 10^8^, 5 × 10^8^ spores/L). Each treatment consisted of three replicates, with 20 insects per replicate.

The milliliter of the prepared solution was placed into a 25 mL centrifuge tube, and rotated to evenly coat the inner wall with a residue film after air drying. Kidney bean pods were cut into 3 cm segments, dipped into the test solution for 15 s, and then air-dried. The treated bean pods were placed into the centrifuge tubes, and 20 *L. pratensis* adults were released into each tube. The insects were maintained in the climate-controlled chamber under the conditions described above. Mortality was recorded at 24, 48, and 72 h post-treatment. Insects were considered dead if no movement or response was observed after gentle probing with a fine brush.

### 2.5. Field Efficacy Trial

The field trial was conducted in the same cotton field as that used for insect collection. The field had level terrain and uniform soil fertility, and the cotton cultivar was Xinluzhong 82. The trial was carried out at the cotton bud stage when the density of *L. pratensis* adults reached 12 individuals per 100 plants. Spraying was performed at 8:00–9:00 a.m. on 17 July under sunny conditions, with a temperature of 23–36 °C, no precipitation, and a northwest wind of Beaufort force 2.

Based on the laboratory toxicity results, four pesticides with relatively high insecticidal activity (abamectin, emamectin benzoate, *B. bassiana* and *M. anisopliae*) were selected for the field efficacy trial. The selection criteria were as follows: (1) high toxicity to *L. pratensis* in laboratory bioassays, with LC_50_ values within the range of field-recommended concentrations; (2) good commercial availability and mature application technology in cotton pest management. Rotenone, matrine and azadirachtin were excluded due to their low toxicity and high required field application dosage.

The 30% dosage reduction rate was selected in accordance with China’s national pesticide reduction and efficiency improvement initiative led by the Ministry of Agriculture and Rural Affairs. On this basis, the specific reduction ratio was determined by referring to relevant studies on adjuvant-enhanced pesticide efficacy in Xinjiang cotton fields, combined with the recommended dosage range of tested pesticides and preliminary bioassay results, to ensure that control efficacy can be maintained with adjuvant assistance while achieving the pesticide reduction target.

Fifteen treatments were arranged in a randomized complete block design with four replications (Table 1). Each plot was 20 m^2^, with a 2 m buffer zone between plots. The spray volume was 30 L/667 m^2^ (approximately 450 L/ha), and the solution was evenly sprayed onto the upper and lower surfaces of cotton leaves. The five-point sampling method was used to investigate the pest population before treatment. Specifically, five sampling points were evenly arranged along the diagonal of each plot, and 10 consecutive cotton plants were investigated at each point, with a total of 50 plants surveyed per plot. The same marked cotton plants were investigated at each observation date to ensure data consistency. The number of surviving *L. pratensis* adults and nymphs on each plant was recorded. At 1, 3, 5, and 7 days after treatment, the number of surviving *L. pratensis* individuals in each plot was recorded. The population decline rate and corrected control efficacy were calculated. Phytotoxicity to cotton plants was observed throughout the trial period.

### 2.6. Data Analysis

All statistical analyses were performed using SPSS 20.0 software. Probit analysis was used to derive toxicity regression equations, median lethal concentration (LC_50_) values, and their 95% confidence intervals.

For field efficacy data, analyses were conducted separately for each sampling date. Residual normality and variance homogeneity were verified via the Shapiro–Wilk test and Levene’s test prior to ANOVA. Given negative control efficacy values in early-stage fungal treatments, arcsine square-root transformation was infeasible, and raw percentage data were analyzed directly. A two-way ANOVA with treatment and block as fixed factors was applied to evaluate treatment effects on control efficacy, followed by Duncan’s multiple range test for post hoc pairwise comparisons (*p* < 0.05).

A mixed-effects model with block as a random factor was considered during study design. However, with only four blocks at a single site, the standard fixed-block randomized complete block ANOVA yields more robust variance estimates, consistent with standard practice for single-site agrochemical field trials.

The formulas for population decline rate and control efficacy are as follows.

Population decline rate = (number of live insects before treatment − number of live insects after treatment)/(number of live insects before treatment) × 100%

Control efficacy = (Population decline rate in treatment group − Population decline rate in control group)/(1 − Population decline rate in control group) × 100%

## 3. Results

### 3.1. Laboratory Bioactivity

**Figure 1:**
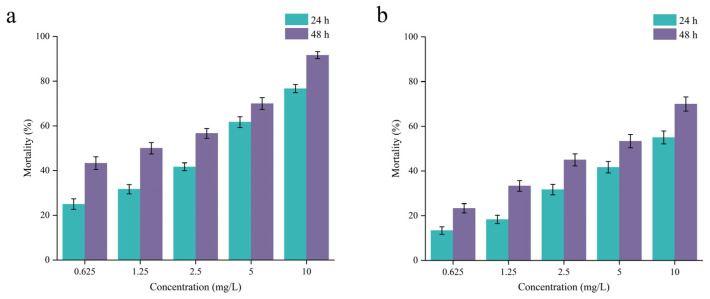
The laboratory toxicity effects of abamectin and emamectin benzoate against *L. pratensis*. (**a**) Abamectin, (**b**) emamectin benzoate at 24 h and 48 h post-treatment. Error bars indicate standard error (SE).

**Figure 2:**
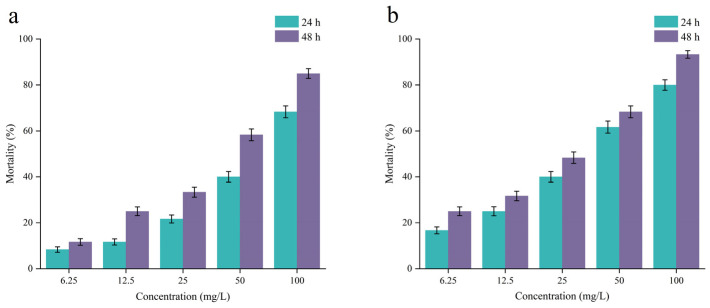
The laboratory toxicity effects of matrine and rotenone against *L. pratensis*. (**a**) Matrine, (**b**) rotenone at 24 h and 48 h post-treatment. Error bars indicate standard error (SE).

**Figure 3:**
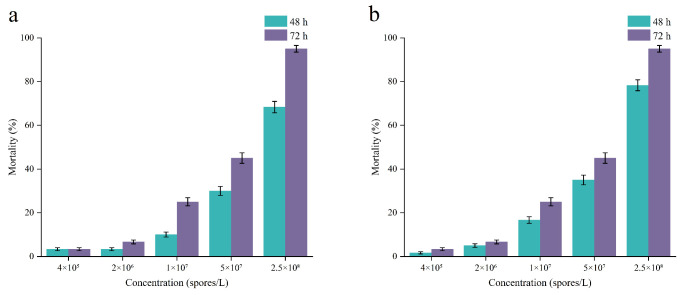
The laboratory toxicity effects of *B. bassiana* and *M. anisopliae* against *L. pratensis*. (**a**) *B. bassiana*, (**b**) *M. anisopliae* at 48 h and 72 h post-treatment. Error bars indicate standard error (SE).

The laboratory toxicity bioassays showed that the mortality of *L. pratensis* reached 91.67% and 70.00% at 48 h after treatment with abamectin and emamectin benzoate at 10 mg/L, respectively (Figure 1). At 48 h after treatment with rotenone and matrine at 100 mg/L, the mortality rates of *L. pratensis* were 85.00% and 93.33%, respectively (Figure 2). Treatment with *M. anisopliae* and *B. bassiana* at a concentration of 2.5 × 10^8^ spores/L resulted in a mortality of 95.00% at 72 h after application (Figure 3). Abamectin and emamectin benzoate exhibited strong and rapid insecticidal activity. The 24 h LC_50_ values were 2.880 mg/L and 7.690 mg/L, and the 48 h LC_50_ values were 1.198 mg/L and 3.424 mg/L, respectively. *M. anisopliae* and *B. bassiana* exhibited slower activity, with 48 h LC_50_ values of 1.52 × 10^8^ spores/L and 1.73 × 10^8^ spores/L, and 72 h LC_50_ values of 9.20 × 10^7^ spores/L and 1.02 × 10^8^ spores/L, respectively (Table 2). Rotenone and matrine were less toxic against *L. pratensis*. Based on their relatively high insecticidal activity in the laboratory bioassays, abamectin, emamectin benzoate, *B. bassiana*, and *M. anisopliae* were selected for subsequent field efficacy evaluations.

### 3.2. Field Efficacy

Abamectin and emamectin benzoate exhibited rapid and high efficacy (Table 3). Abamectin (4.20 g a.i./hm^2^) combined with d-limonene or mineral oil provided 63.75–89.24% control efficacy over 1 to 7 days after treatment. No significant difference was detected between these reduced-rate adjuvant mixtures and the full-rate abamectin single-agent treatment (*p* > 0.05). The control efficacy remained above 60% at 7 days after treatment. Among all abamectin-related treatments, abamectin (4.20 g a.i./hm^2^) plus mineral oil showed the highest efficacy (89.24%) at 3 days after treatment, but the difference was not significant compared with the full-rate abamectin treatment (*p* > 0.05). At 5 days post-treatment, the treatment of emamectin benzoate (7.78 g a.i./hm^2^) plus mineral oil achieved 86.17% control efficacy, which was numerically higher than that of the full-rate emamectin benzoate alone, with no significant difference detected (*p* > 0.05).

*B. bassiana* and *M. anisopliae* exhibited slow but persistent activity (Table 4). Compared with the full-rate single-agent fungal treatments, the reduced-rate adjuvant-amended fungal treatments showed numerically earlier efficacy onset, but the difference did not reach statistical significance at most observation time points (*p* > 0.05). The enhancement effect of d-limonene was numerically stronger than that of mineral oil, but the difference was not statistically significant at most observation dates (*p* > 0.05). The treatment of *B. bassiana* (6.30 × 10^12^ spores/hm^2^) combined with d-limonene provided 10.40–10.84% efficacy at 1 to 3 days post-treatment. The efficacy was numerically higher than that of the full-rate fungus alone and the mineral oil mixture, but most of the differences did not reach statistical significance (*p* > 0.05). The treatment of *M. anisopliae* (7.35 × 10^12^ spores/hm^2^) plus d-limonene provided 17.73–70.50% efficacy at 1–7 days. Its efficacy was comparable to that of the mineral oil mixture (*p* > 0.05), and significantly higher than that of the full-rate fungus alone at 7 days post-treatment (*p* < 0.05). No phytotoxicity symptoms were observed on cotton plants during the trial.

**Figure 4:**
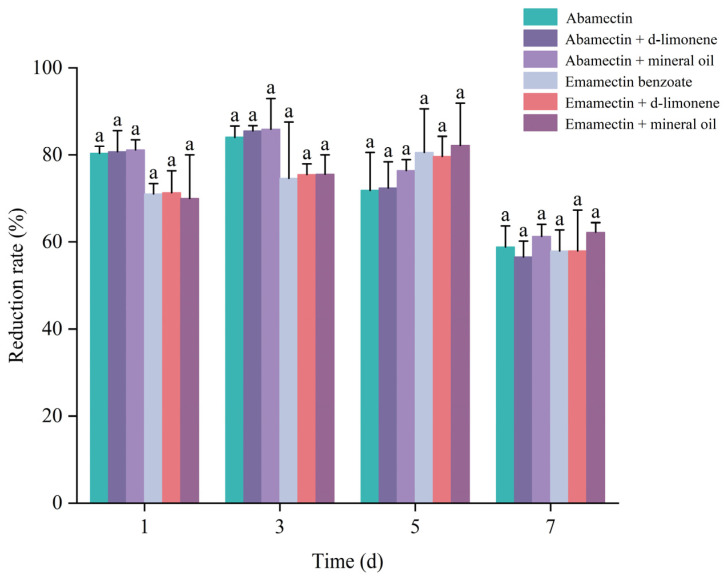
Population reduction dynamics of *L. pratensis* under abamectin and emamectin benzoate treatments with or without adjuvants. Error bars = SE of four replicates. Different lowercase letters indicate significant differences among treatments at the same time point (Duncan’s test, *p* < 0.05).

**Figure 5:**
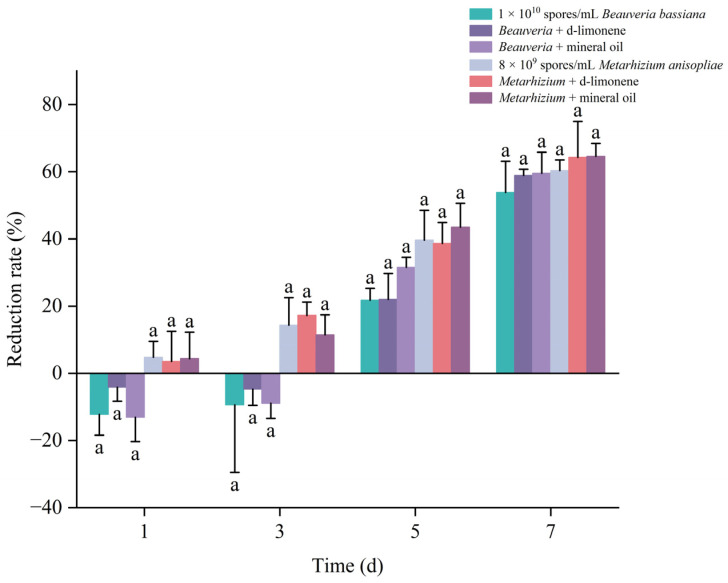
Population reduction dynamics of *L. pratensis* induced by *B. bassiana*, *M. anisopliae* and their adjuvant combinations. Error bars = SE of four replicates. Different lowercase letters indicate significant differences among treatments at the same time point (Duncan’s test, *p* < 0.05).

**Figure 6:**
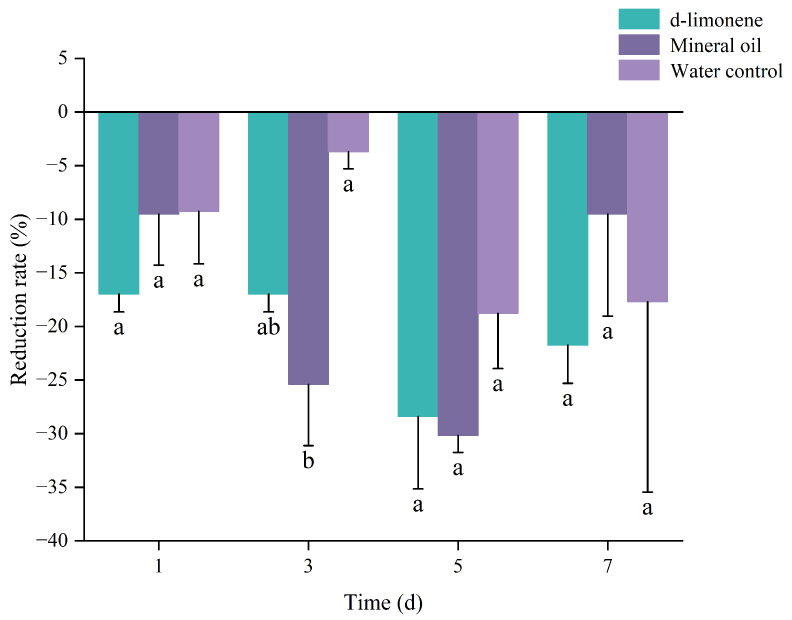
Population reduction dynamics of *L. pratensis* treated with d-limonene, mineral oil and water control. Error bars = SE of four replicates. Different lowercase letters indicate significant differences among treatments at the same time point (Duncan’s test, *p* < 0.05).

The abamectin and emamectin benzoate treatments both exhibited rapid and high initial efficacy; specifically, abamectin-related formulations peaked at 3 days post-treatment, with the abamectin plus mineral oil mixture achieving the numerically highest reduction rate of 89.24%, whereas emamectin benzoate treatments displayed a slightly delayed but more persistent efficacy, with their reduction rates peaking at 5 days post-treatment, notably reaching 86.17% for the emamectin benzoate plus mineral oil cohort, with no significant difference from the full-rate treatment (*p* > 0.05) (Figure 4). In contrast, the entomopathogenic fungal treatments of *B. bassiana* and *M. anisopliae* exhibited a slow but persistent efficacy profile; although their early-stage efficacy at 1 to 3 days post-treatment was minimal, the incorporation of adjuvants, particularly d-limonene, numerically accelerated efficacy onset, triggering a consistent upward trend across all fungal cohorts from 3 days post-treatment onward, with no significant difference in efficacy between the two adjuvant treatments at most time points (*p* > 0.05), and reaching maximum reduction rates of 50–75% at 7 days post-treatment (Figure 5). Furthermore, standalone treatments of d-limonene, mineral oil, and the water control revealed that throughout the observation period, the insect reduction rates for these three groups consistently remained within the negative domain, indicating an increasing trend in the field populations of *L. pratensis* and thereby demonstrating that these two adjuvants exert no direct lethal or suppressive control efficacy when applied alone (Figure 6).

## 4. Discussion and Conclusions

Long-term and excessive use of chemical pesticides has resulted in increased pest resistance, non-target effects, and agroecosystem contamination, posing threats to sustainable agricultural production; these ecological risks have been extensively documented in previous studies [10]. Biopesticides feature high target specificity, environmental safety, and low resistance risk, and their importance in integrated pest management (IPM) programs has been increasingly recognized [11], with widely demonstrated potential for sustainable pest control [12]. This study evaluated four commonly used microbial pesticides—abamectin, emamectin benzoate, *B. bassiana*, and *M. anisopliae*—and assessed the potential of adjuvant-amended combinations for *L. pratensis* management.

Laboratory and field results revealed distinct differences in action speed and control efficacy among the tested agents. Abamectin and emamectin benzoate, as macrocyclic lactone insecticides, exhibited rapid contact and stomach toxicity against *L. pratensis* under laboratory conditions. These findings align with previous reports of emamectin benzoate’s rapid insecticidal activity against *Helicoverpa armigera* [13], *Spodoptera litura*, *Ostrinia furnacalis* [14,15], and *Spodoptera frugiperda* [16], and field studies have further confirmed their strong short-term suppression of lepidopteran pest populations [17]. In contrast, *B. bassiana* and *M. anisopliae* showed limited rapid efficacy when applied alone, with relatively low field performance in the first 1–2 days post-treatment. This is consistent with the inherent slow mode of action of entomopathogenic fungi, which require completion of the full infection cycle including spore germination, host penetration, and colonization [18], and matches previous reports of gradual pathogenic effects on *Aedes aegypti* larvae [19]. Despite slower onset, both fungi showed considerable long-term suppression potential due to their strong infectivity. Previous studies have reported up to 100% corrected mortality of *M. anisopliae* against *Halyomorpha halys* [20], and high pathogenicity of *B. bassiana* against *Sipha flava* and *Ceratovacuna lanigera* [21,22]. As important biocontrol agents widely applied in agricultural and forest pest management [11], these fungi can effectively reduce pod damage by *H. armigera* in chickpea systems [23] and deliver consistent field performance against lepidopteran pests such as *Pieris rapae* and *Plutella xylostella* [24].

Adjuvant application represents an effective strategy to address the slow-onset limitation of microbial pesticides, especially entomopathogenic fungi. Plant essential oils and mineral oils can enhance insecticidal performance by improving wettability, spreading, and cuticular penetration of active ingredients [25]; mineral oil in particular has been reported to deliver notable adjuvant effects when combined with insecticides, boosting pest suppression even at low application rates [26]. This study confirmed that 30% reduced rates of abamectin and emamectin benzoate, when combined with d-limonene or mineral oil, maintained control efficacy against *L. pratensis* comparable to full-rate applications, indicating that pesticide input can be reduced without compromising control performance. It has been reported that d-limonene can disrupt the insect cuticular wax layer, which may facilitate fungal spore adhesion and infection; it also exhibits weak direct insecticidal activity, which may complement the pathogenic action of fungal infection [27,28]. Previous studies have also shown that adjuvants can form a stable microenvironment around fungal spores, alleviating UV and drought-induced viability loss and prolonging field persistence [29], which may partially explain the improved efficacy observed in this study, though the specific mechanism requires further targeted verification. The differential enhancement of the two adjuvants is related to their distinct action properties. For entomopathogenic fungi, d-limonene showed slightly better performance, which may be attributed to its strong wax-dissolving effect that is more prominent under the low-humidity conditions of Xinjiang and for the smooth cuticle of *L. pratensis* [30]. This also explains the discrepancy with some studies reporting better fungal enhancement by mineral oil, which may stem from differences in target pests, fungal strains, and ambient humidity. The varied response of the two fungal species may be associated with their distinct conidial surface properties: *M. anisopliae* conidia are relatively hydrophobic, so adjuvants more obviously improve their wettability and adhesion on hydrophobic cotton leaves, while *B. bassiana* conidia with stronger hydrophilicity gain relatively less benefit. For abamectin and emamectin benzoate, the core limiting factor for field efficacy in Xinjiang is rapid photodegradation under intense UV radiation. It is speculated that mineral oil may form a long-lasting protective film on the leaf surface to delay active ingredient degradation, which is why it slightly improves the persistence of these antibiotic insecticides [31]. Overall, this adjuvant-assisted strategy supports the development of environmentally sustainable pest management.

The four microbial pesticides evaluated in this study all show considerable potential for *L. pratensis* management: microbial-derived insecticides excel in rapid efficacy, while entomopathogenic fungi have advantages in persistent control. Under the tested conditions, combining the four agents with green adjuvants (d-limonene and mineral oil) can effectively compensate for the slow onset of single fungal agents and maintain or improve overall field efficacy under 30% dosage reduction, providing a feasible regional reference for reducing pesticide input and supporting integrated *L. pratensis* management in Xinjiang cotton fields under similar arid climatic conditions.

Several limitations of this study should be acknowledged. First, the field trial did not include reduced-rate pesticide treatments without adjuvants, so the independent contribution of adjuvants cannot be completely separated from the effect of dosage reduction. The observed efficacy of the mixtures is the combined result of dosage adjustment and adjuvant addition, and a true synergistic interaction cannot be confirmed by the current experimental design. Second, no formal synergy interaction analysis (such as Colby method, co-toxicity coefficient or synergy factor) was performed in this study, so the efficacy improvement of the mixtures cannot be defined as synergistic effect in a strict sense. Third, the field trial was conducted at a single location during a single growing season, and the efficacy may vary under different climatic conditions and pest population densities. Multi-location and multi-season trials are needed to verify the stability of the strategy.

## Figures and Tables

**Table 1:** Field Efficacy Test Treatments.

Test Insecticides	Application Dosage
10% Abamectin SC	6.00 g a.i./hm^2^ (recommended full rate)
	4.20 g a.i./hm^2^ (30% reduced rate) + 225.00 mL/hm^2^ d-Limonene
	4.20 g a.i./hm^2^ (30% reduced rate) + 225.00 mL/hm^2^ Mineral Oil
1.14% Emamectin Benzoate ME	11.12 g a.i./hm^2^ (recommended full rate)
	7.78 g a.i./hm^2^ (30% reduced rate) + 225.00 mL/hm^2^ d-Limonene
	7.78 g a.i./hm^2^ (30% reduced rate) + 225.00 mL/hm^2^ Mineral Oil
*B. bassiana* ZJU435 WP (1 × 10^10^ spores/mL)	9 × 10^12^ spores/hm^2^ (recommended full rate)
	6.3 × 10^12^ spores/hm^2^ (30% reduced rate) + 225.00 mL/hm^2^ d-Limonene
	6.3 × 10^12^ spores/hm^2^ (30% reduced rate) + 225.00 mL/hm^2^ Mineral Oil
*M. anisopliae* CQMa421 (8 × 10^9^ spores/mL)	1.05 × 10^13^ spores/hm^2^ (recommended full rate)
	7.35 × 10^12^ spores/hm^2^ (30% reduced rate) + 225.00 mL/hm^2^ d-Limonene
	7.35 × 10^12^ spores/hm^2^ (30% reduced rate) + 225.00 mL/hm^2^ Mineral Oil
d-Limonene	225.00 mL/hm^2^
Mineral Oil	225.00 mL/hm^2^
Clean Water Control	Same volume of water (450 L/hm^2^)

Note: The spray volume for all treatments was 450 L/hm^2^. The clean water control was applied with the same volume of water as the carrier control, without any pesticide or adjuvant.

**Table 2:** Laboratory toxicity of seven microbial-derived pesticides against *L. pratensis* adults (24, 48, and 72 h).

Test Agent	Time/h	Regression Equation (*y* =)	LC_50_/[mg (Spores)·L^−1^]	95% Confidence Interval	*df*	*x* ^2^
Abamectin	24	−0.54 + 1.19*x*	2.880	2.152~3.954	3	1.446
	48	−0.10 + 1.20*x*	1.198	0.156~2.373	3	5.374
	72	/	/	/	/	/
Emamectin benzoate	24	−0.93 + 1.05*x*	7.690	5.200~14.983	3	0.233
	48	−0.53 + 1.00*x*	3.424	2.440~5.199	3	0.379
	72	/	/	/	/	/
Rotenone	24	−2.27 + 1.48*x*	35.002	27.623~45.985	3	2.526
	48	−2.31 + 1.76*x*	21.337	11.716~36.478	3	5.690
	72	/	/	/	/	/
Matrine	24	−2.79 + 1.55*x*	62.471	48.547~88.027	3	2.887
	48	−2.69 + 1.77*x*	33.793	27.536~42.415	3	3.248
	72	/	/	/	/	/
*M. anisopliae* CQMa421	24	/	/	/	/	/
	48	−1.46 + 0.95*x*	1.52 × 10^8^	6.98 × 10^7^~5.39 × 10^8^	3	18.168
	72	−1.25 + 1.21*x*	9.20 × 10^7^	3.21 × 10^7^~5.42 × 10^8^	3	18.441
*B. bassiana* ZJU435	24	/	/	/	/	/
	48	−1.53 + 0.85*x*	1.73 × 10^8^	8.98 × 10^7^~5.88 × 10^8^	3	12.696
	72	−1.33 + 1.12*x*	1.02 × 10^8^	4.76 × 10^7^~2.48 × 10^8^	3	11.736

**Table 3:** Field efficacy of abamectin and emamectin benzoate at 30% reduced dosage mixed with two adjuvants against *L. pratensis*.

Treatment	Application Rate (g a.i./hm^2^)	Adjuvant (mL/hm^2^)	1 d	3 d	5 d	7 d
			Control Efficacy/%	Control Efficacy/%	Control Efficacy/%	Control Efficacy/%
Abamectin	6.00	/	82.06 ± 1.04 a	84.68 ± 0.71 a	76.60 ± 7.12 a	64.03 ± 4.99 a
Abamectin + d-Limonene	4.2	225.00	83.56 ± 4.11 a	87.64 ± 0.85 a	78.15 ± 5.15 a	64.14 ± 3.64 a
Abamectin + mineral oil	4.2	225.00	82.69 ± 2.41 a	89.24 ± 5.42 a	81.83 ± 2.13 a	63.74 ± 5.23 a
Emamectin benzoate	11.12	/	73.57 ± 1.09 a	73.3 ± 15.93 a	83.99 ± 8.43 a	61.94 ± 8.02 a
Emamectin + d-Limonene	7.78	225.00	75.34 ± 4.66 a	78.99 ± 2.3 a	83.83 ± 4.37 a	65.45 ± 7.84 a
Emamectin + mineral oil	7.78	225.00	72.92 ± 8.36 a	80.52 ± 3.31 a	86.17 ± 7.53 a	64.69 ± 4.69 a

Note: Data are means ± SE. Different lowercase letters in the same column indicate significant difference at *p* < 0.05.

**Table 4:** Field efficacy of entomopathogenic fungi at 30% reduced dosage mixed with two adjuvants against *L. pratensis*.

Treatment	Application Rate (spores/hm^2^)	Adjuvant (mL/hm^2^)	1 d	3 d	5 d	7 d
			Control Efficacy/%	Control Efficacy/%	Control Efficacy/%	Control Efficacy/%
1 × 10^10^ spores/mL *B. bassiana*	9 × 10^12^	/	−3.17 ± 7.70 b	−4.50 ± 7.17 a	34.11 ± 1.35 a	61.37 ± 3.42 a
*Beauveria* + d-Limonene	6.3 × 10^12^	225.00	10.84 ± 4.69 ab	10.4 ± 4.35 a	38.90 ± 7.18 a	66.13 ± 2.37 a
*Beauveria* + mineral oil	6.3 × 10^12^	225.00	−3.13 ± 3.13 b	13.1 ± 0.60 a	47.42 ± 2.07 a	63.23 ± 4.26 a
8 × 10^9^ spores/mL *M. anisopliae*	1.05 × 10^13^	/	12.86 ± 1.43 ab	11.90 ± 20.34 a	48.48 ± 9.10 a	65.48 ± 2.98 a
*Metarhizium* + d-Limonene	7.35 × 10^12^	225.00	17.73 ± 6.50 a	29.32 ± 2.73 a	52.17 ± 4.53 a	70.50 ± 9.08 a
*Metarhizium* + mineral oil	7.35 × 10^12^	225.00	12.77 ± 5.39 ab	28.66 ± 8.10 a	56.45 ± 5.90 a	66.83 ± 5.64 a

Note: Data are means ± SE. Different lowercase letters in the same column indicate significant difference at *p* < 0.05. Negative control efficacy values indicate that the pest population in the treatment group increased faster than that in the clean water control at the early stage, which is mainly caused by the slow onset of entomopathogenic fungi and the natural growth of *L. pratensis* population in the field.

## Data Availability

The data presented in this study are available upon request from the corresponding author.

## Abbreviations

The following abbreviations are used in this manuscript:

SC	suspension concentrate
ME	microemulsion
WP	wettable powder
a.i.	active ingredient
hm^2^	hectare
